# Supplementary material for: Predicting postoperative peritoneal metastasis in gastric cancer with serosal invasion using a collagen nomogram
Source: Nat Commun. 2021 Jan 8;12:179. doi: 10.1038/s41467-020-20429-0 (PMC7794254; doi:10.1038/s41467-020-20429-0)
Supplement: Supplementary file 1 — Supplementary Information [file 41467_2020_20429_MOESM1_ESM.pdf]

Supplementary Information

Predicting postoperative peritoneal metastasis in gastric cancer with serosal invasion using a collagen nomogram

Authors: Dexin Chen, Zhangyuanzhu Liu, Wenju Liu, Meiting Fu, Wei Jiang, Shuoyu Xu, Guangxing Wang, Feng Chen, Jianping Lu, Hao Chen, Xiaoyu Dong, Guoxin Li, Gang Chen, Shuangmu Zhuo and Jun Yan

Supplementary Note

Supplementary Note 1

Collagen signature = 0.48748321

+ 0.02321756 * Mean of cross-link density

- 0.08538481 * Gabor_scale1_orientation2

- 0.37606065 * Gabor_scale1_orientation3

- 0.10427976 * Gabor_scale1_orientation4

Supplementary Figures

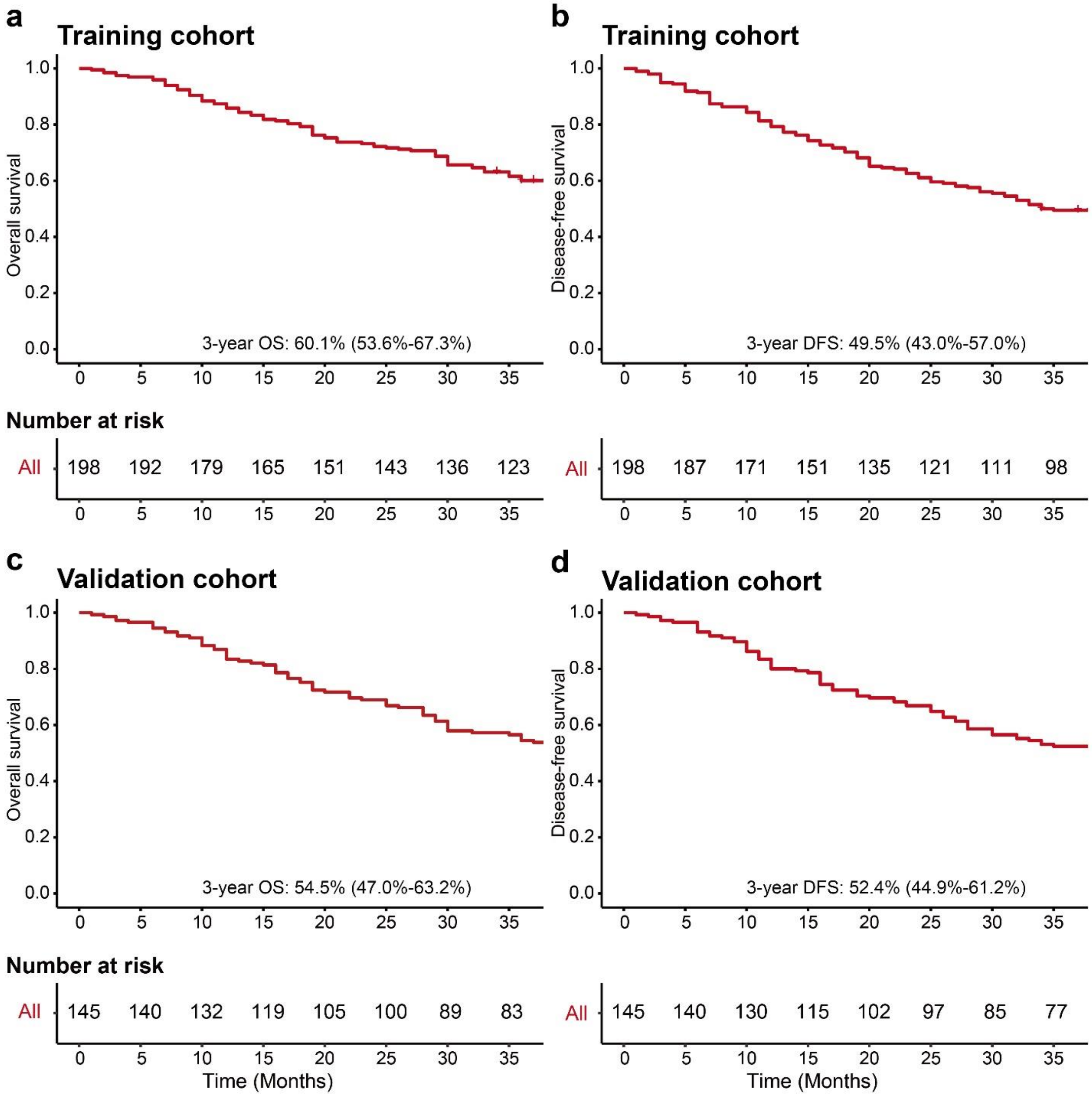

Supplementary Figure 1. Kaplan-Meier survival analysis of the training and validation cohorts. **(a)** The OS curve in the training cohort. **(b)** The DFS curve in the training cohort. **(c)** The OS curve in the validation cohort. **(d)** The DFS curve in the validation cohort. *Abbreviations:* OS, overall survival; DFS, disease-free survival.

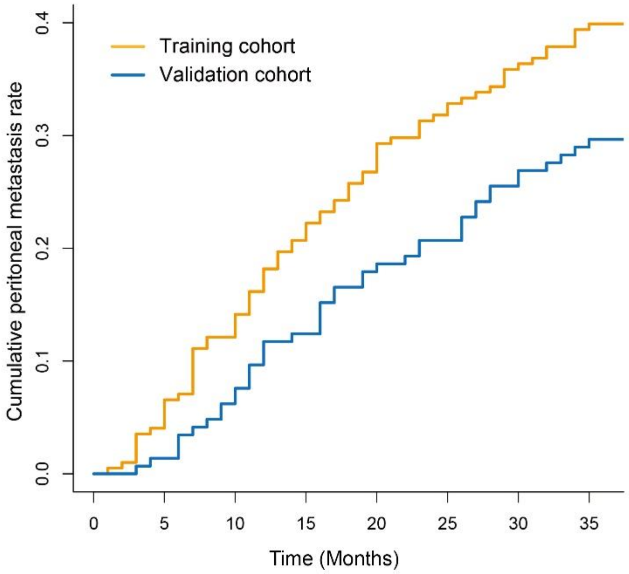

Supplementary Figure 2. Cumulative peritoneal metastasis rate in the training and validation cohorts. The yellow line indicates the cumulative peritoneal metastasis rate in the training cohort, and the blue line indicates the cumulative peritoneal metastasis rate in the validation cohort.

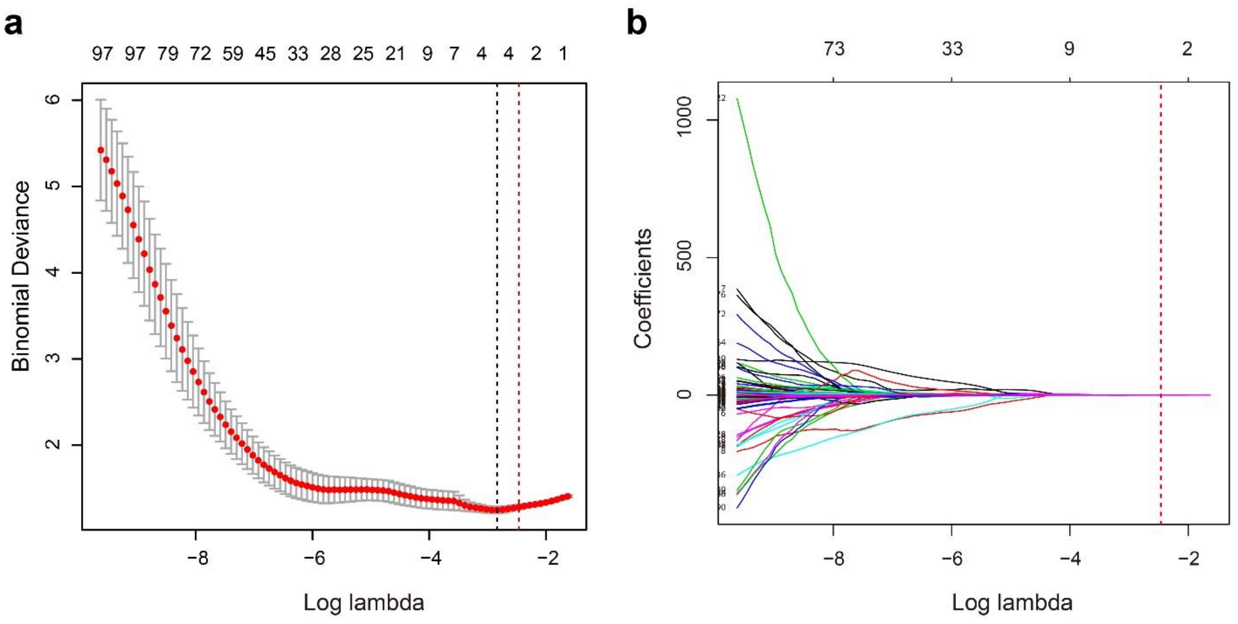

Supplementary Figure 3. Collagen features selection using the LASSO regression model. **(a)** Tuning parameter (λ) selection in the LASSO method using five-time cross-validation. Solid vertical lines represent the binomial deviance ± SE. Black and red dotted vertical lines were drawn at the optimal values by the minimum criteria and the 1-SE criteria, respectively. A λ value of 0.08514, with log (λ) = -2.463, was chosen by five-fold cross-validation via 1-SE criteria. The centre red points of the solid vertical lines represent the mean of binomial deviance under different λ values, and the error bars indicate the SE. **(b)** The LASSO coefficient profiles of the 146 collagen features at different tuning parameters (λ) according to 3-year disease-free survival in the training cohort, and a red dotted vertical line was drawn at log (λ) = -2.463. The corresponding 4 nonzero coefficients resulting from λ were acquired. *Abbreviations:* LASSO, least absolute shrinkage and selection operator; SE, standard error.

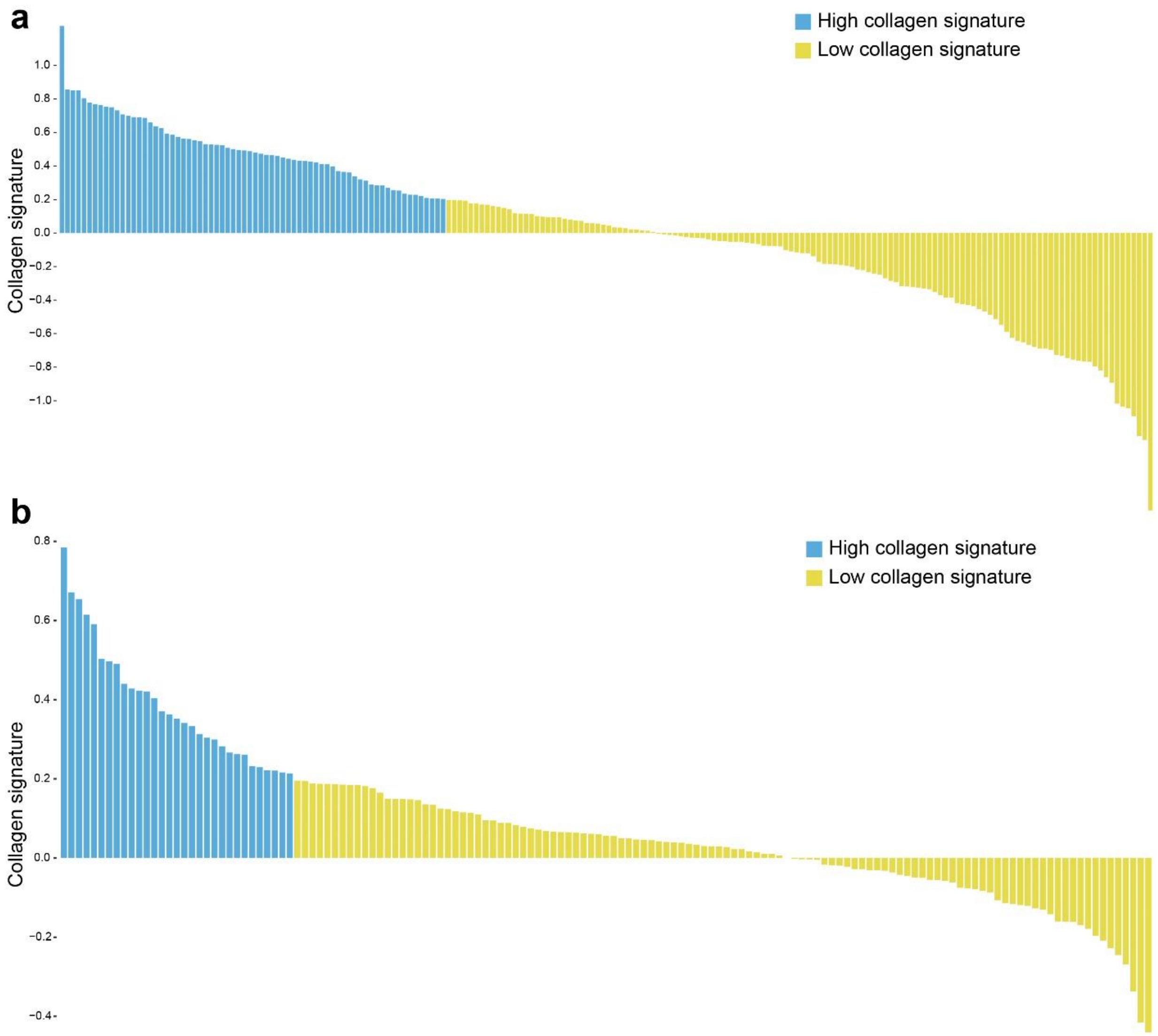

Supplementary Figure 4. Distribution of the collagen signature in the training and validation cohorts. **(a)** The distribution of the collagen signature in the training cohort, with 70 patients classified into the high collagen signature group and 118 patients classified into the low collagen signature group based on a cutoff value of 0.2. **(b)** The distribution of the collagen signature in the validation cohort, with 31 patients classified into the high collagen signature group and 114 patients classified into the low collagen signature group based on a cutoff value of 0.2.

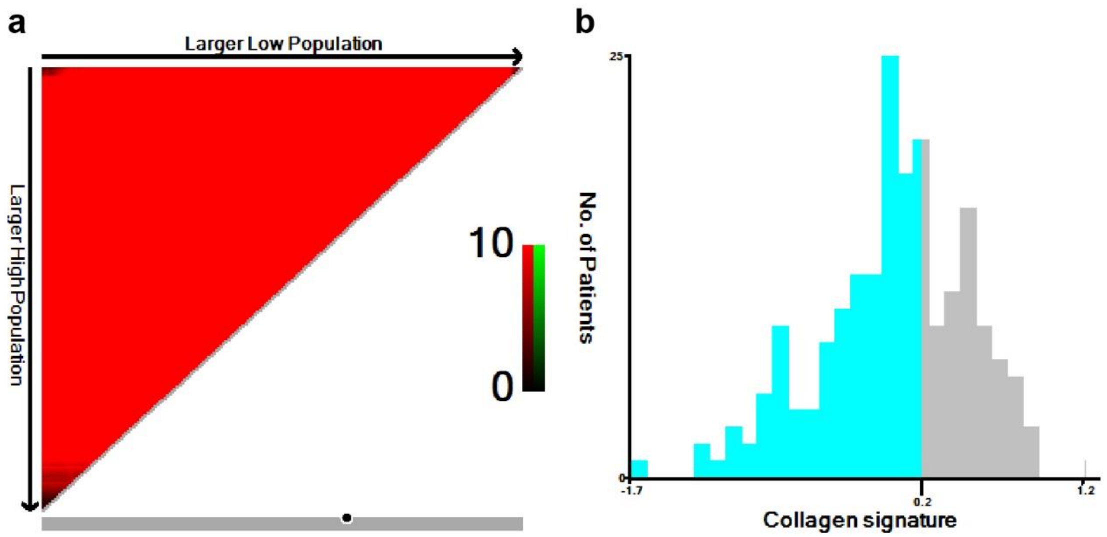

Supplementary Figure 5. X-tile plots of the collagen signature with the cutoff value in the training cohort. **(a)** The colours in the plot represent the strength of the association at each division, ranging from low (black) to high (bright red or green). Red represents the inverse association between the collagen signature and survival, whereas green represents a positive association. The *x*-axis represents all potential cutoff points, from low to high (left to right), that define a low subset, whereas the *y*-axis represents cutoff points from high to low (top to bottom) that define a high subset. The optimum cutoff point is highlighted by the black dot on the *x*-axis. **(b)** The cutoff value of the collagen signature and the numbers of patients in subgroups.

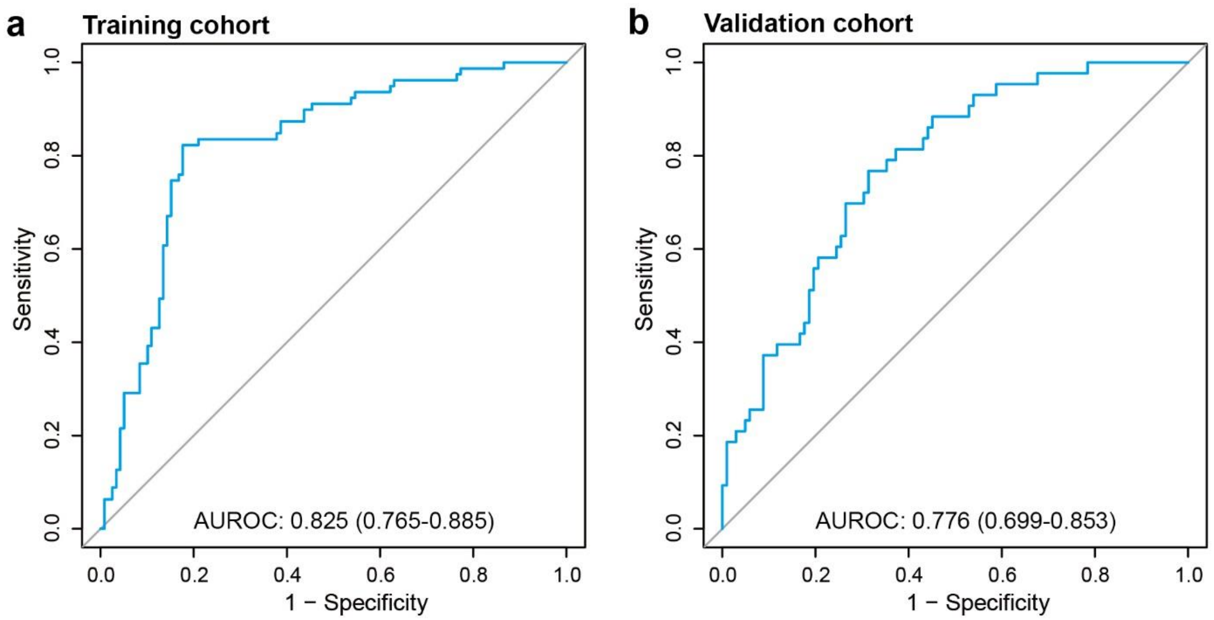

Supplementary Figure 6. The 3-year time-dependent ROC curves of the nomogram in training and validation cohorts. *Abbreviations:* AUROC, area under the receiver operating characteristic curve; ROC, receiver operating characteristic.

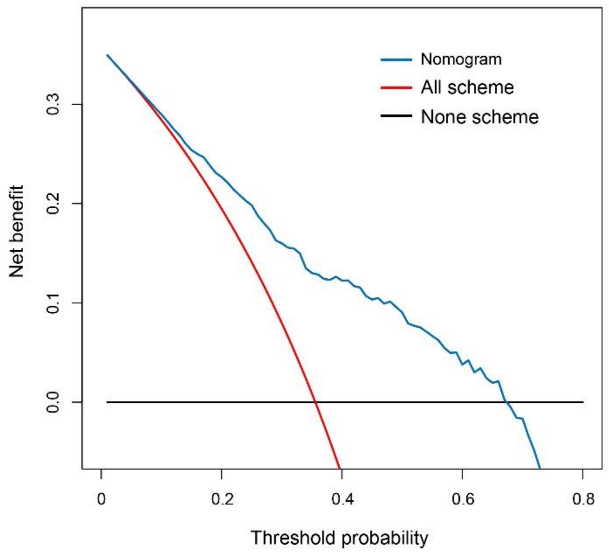

Supplementary Figure 7. Decision curve analysis for the nomogram. The blue line represents the competing-risk nomogram, the red line represents the treat-all scheme, and the black line represents the treat-none scheme.

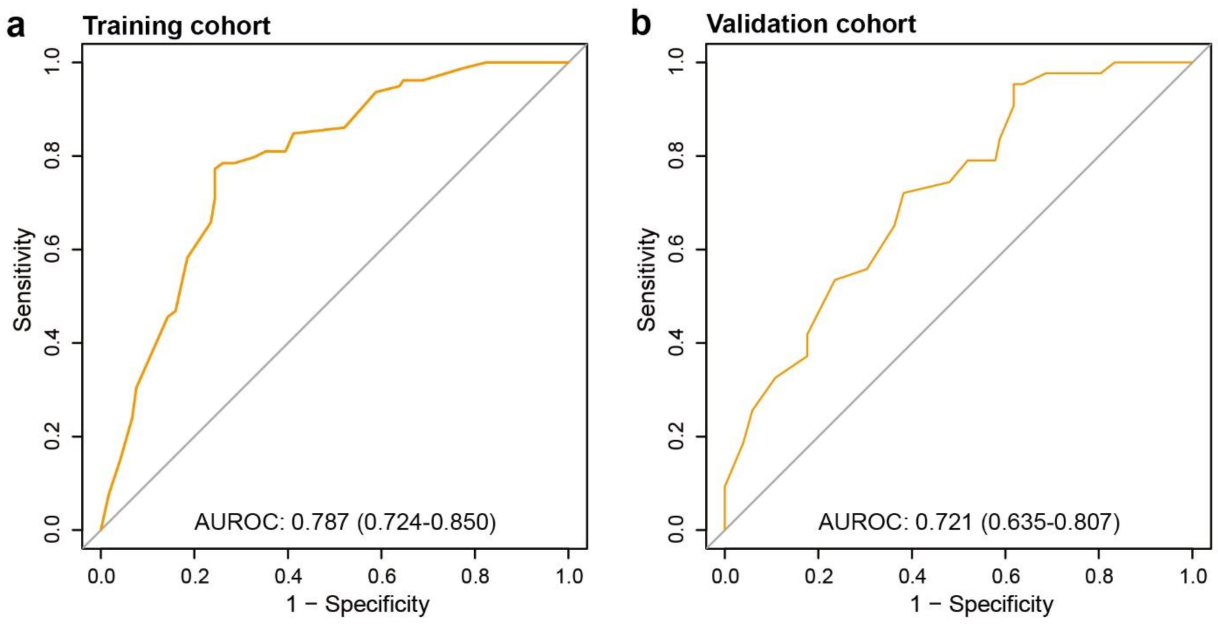

Supplementary Figure 8. The 3-year time-dependent ROC curves of the clinicopathological model in the **(a)** training and **(b)** validation cohorts. *Abbreviations:* AUROC, area under the receiver operating characteristic curve; ROC, receiver operating characteristic.

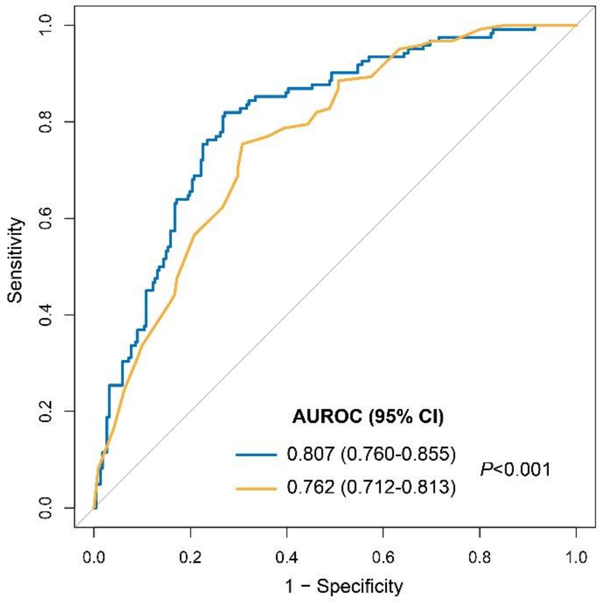

Supplementary Figure 9. The ROC curves comparison between the nomogram and clinicopathological model. The blue line indicates the nomogram based on the collagen signature, and the yellow line indicates the clinicopathological model. A two-sided Delong test is used for comparison between the two models. *Abbreviations:* AUROC, area under the receiver operating characteristic curve; ROC, receiver operating characteristic.

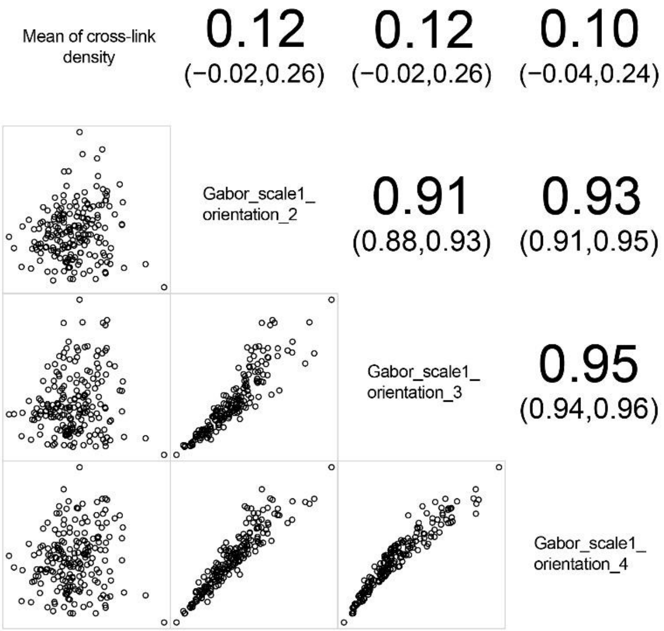

Supplementary Figure 10. Correlation analysis between the selected features. The bottom left plots indicate the scatterplots between two features in turn. The top right values denote Pearson correlation coefficients with corresponding confidence internals, and values closer to 1 identify a better correlation.

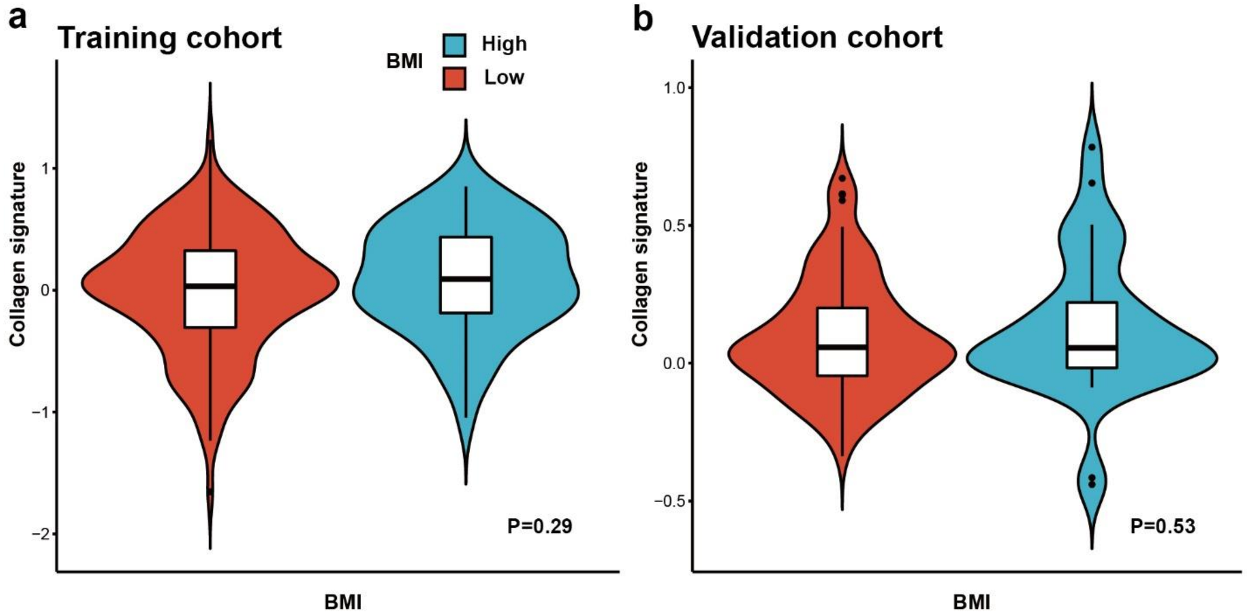

Supplementary Figure 11. Collagen signature distribution based on BMI in the **(a)** training and **(b)** validation cohorts. In training cohort, n=151 and 47 patients for low BMI and high BMI subgroups, respectively. In validation cohort, n=108 and 37 patients for low BMI and high BMI subgroups, rescpectively. In violin plots, the centre lines are median, the upper and lower bounds of boxes indicate first and third quartiles, the whiskers mean the 1.5 times of interquartile range, and the upper and lower tails represent the maxima and minima, respectively. A two-sided Mann-Whitney *U* test is used for comparisons between the two subgroups. *Abbreviations:* BMI, body mass index.

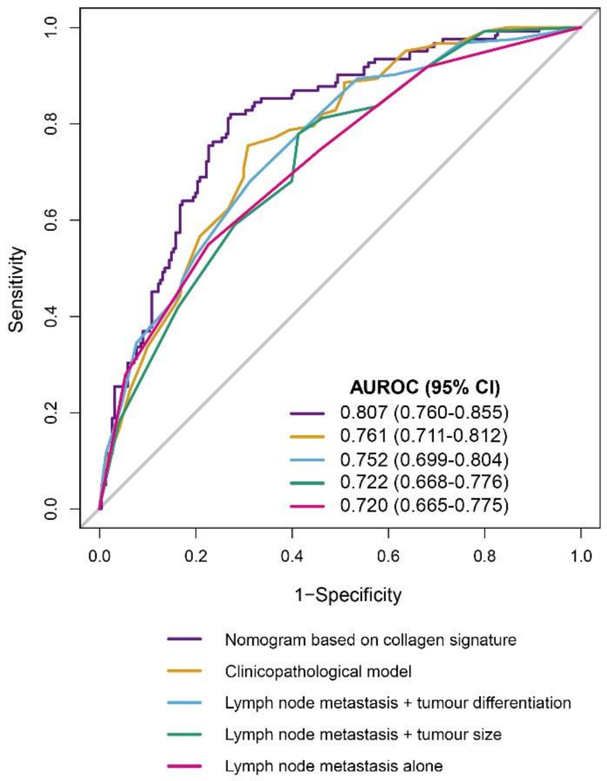

Supplementary Figure 12. Prediction performances for peritoneal metastasis with lymph node metastasis and different combinations of variables. *Abbreviations:* AUROC, area under the receiver operating characteristic curve.

Supplementary Tables

Supplementary Table 1. Summarization of competing events in the training and validation cohorts

Type of competing event	Training cohort	Validation cohort
**Death, no.**	2	3
**Local recurrence, no.**	3	6
**Distant metastasis, no.**	-	-
Liver	7	8
Lung	5	4
Lymph node	3	1
Bone	1	3
Brain	-	1
**Total, no.**	21	26

Supplementary Table 2. Clinical characteristics of patients according to the collagen signature

Variable	Training cohort		*P* value	Validation cohort		*P* value
	High (n=70)	Low (n=128)		High (n=31)	Low (n=114)	
**Age, median (IQR), years**	57.5 (47.75 to 64)	56.5 (47.25 to 62)	0.39	58 (52 to 62)	56.5 (51.75 to 66)	0.51
**Sex, no. (%)**						
Male	49 (70.0)	88 (68.8)	0.86	20 (71.1)	78 (68.4)	0.85
Female	21 (30.0)	40 (31.2)		11 (28.9)	36 (31.6)	
**BMI, no. (%)**						
≥24 kg/m^2	20 (28.6)	27 (21.1)	0.24	20 (71.1)	88 (77.2)	0.23
<24 kg/m^2	50 (71.4)	101 (78.9)		11 (28.9)	26 (22.8)	
**CEA, no. (%)**						
Normal	47 (67.1)	93 (72.7)	0.42	23 (74.2)	83 (72.8)	0.99
Elevated	23 (32.9)	35 (27.3)		8 (25.8)	31 (27.2)	
**CA 19-9, no. (%)**						
Normal	52 (74.3)	98 (76.6)	0.72	23 (74.2)	93 (81.6)	0.51
Elevated	18 (25.7)	30 (23.4)		8 (25.8)	21 (18.4)	
**Tumour location**						
Cardia of the stomach	14 (20.7)	27 (21.1)		10 (33.3)	34 (29.8)	
Body of the stomach	29 (41.4)	21 (16.4)	<0.001	7 (22.6)	29 (25.4)	0.94
Antrum of the stomach	27 (38.6)	80 (62.5)		14 (45.1)	51 (44.8)	
**Tumour size, no. (%)**						
≥4 cm	49 (70.0)	65 (50.8)	0.009	20 (64.5)	73 (64.0)	0.99
<4 cm	21 (30.0)	63 (49.2)		11 (35.5)	41 (36.0)	
**Lauren classification, no. (%)**						
Intestinal	26 (37.1)	45 (35.2)	0.90	12 (38.7)	49 (43.0)	0.82
Diffuse or mixed	44 (62.9)	83 (64.8)		19 (61.3)	65 (57.0)	
**Differentiation status, no. (%)**						
Well and moderate	14 (20.0)	36 (28.1)		11 (35.4)	35 (30.7)	
Poor	36 (51.4)	59 (46.1)	0.45	10 (32.3)	50 (43.9)	0.50
Undifferentiated	20 (28.6)	33 (25.8)		10 (32.3)	29 (25.4)	
**Lymph node metastasis, no. (%)**						
N0	9 (12.9)	43 (33.6)		6 (19.4)	23 (20.2)	
N1	15 (21.4)	30 (23.4)		5 (16.1)	20 (17.5)	
N2	15 (21.4)	20 (15.6)	0.006	5 (16.1)	35 (30.7)	0.25
N3a	15 (21.4)	23 (18.0)		8 (25.8)	25 (21.9)	
N3b	16 (22.9)	12 (9.4)		7 (22.6)	11 (9.7)	
**Chemotherapy, no. (%)**						
Yes	46 (65.7)	98 (76.6)	0.10	21 (67.7)	72 (63.2)	0.79
No	24 (34.3)	30 (23.4)		10 (32.3)	42 (36.8)	

The comparisons of age between two subgroups are performed using a two-sided Mann-Whitney *U* test, and the rest variables are compared using a two-sided χ^2 or Fisher's exact test.
Abbreviations: BMI, body mass index; CA, carbohydrate antigen CEA, carcinoembryonic antigen; IQR, interquartile range.

Supplementary Table 3. Performance evaluation of the nomogram

Index	Training cohort	Validation cohort	Total cohort
Threshold	0.3913	0.3913	0.3913
Sensitivity, %	82.3 (73.4-91.4)	81.4 (69.8-93.0)	82.0 (74.6-88.6)
Specificity, %	82.4 (74.8-89.1)	60.8 (51.0-69.6)	72.4 (66.1-78.3)
Accuracy, %	82.3 (76.8-87.4)	66.9 (59.3-74.5)	75.8 (71.4-80.2)
Negative predictive value, %	87.7 (82.5-92.8)	88.9 (82.2-95.2)	88.0 (83.7-92.2)
Positive predictive value, %	75.6 (68.5-83.3)	46.8 (40.2-54.3)	62.1 (56.7-66.8)

Supplementary Table 4. Univariate and multivariate Fine-Gray regression without collagen signature

Variable	Univariate analysis		Multivariate analysis	
	SHR (95% CI)	***P* value**	SHR (95% CI)	***P* value**
**Age**	0.99 (0.98-1.02)	0.62		
**Sex** (Female vs. Male)	0.87 (0.53-1.43)	0.58		
**BMI (**≥24 kg/m^2 vs. <24 kg/m^2**)**	1.14 (0.69-1.89)	0.61		
**CEA** (Elevated vs. Normal)	1.08 (0.67-1.73)	0.76		
**CA 19-9** (Elevated vs. Normal)	1.58 (0.98-2.55)	0.064		
**Tumour location**		0.56		
Antrum of the stomach	Reference	>0.99		
Body of the stomach	1.32 (0.80-2.19)	0.28		
Cardia of the stomach	1.07 (0.60-1.90)	0.82		
**Tumour size** (≥4 cm vs. <4 cm)	3.32 (1.99-5.52)	<0.001	2.70 (1.57-4.63)	<0.001
**Lauren classification (**Diffuse or mixed vs. Intestinal**)**	1.30 (0.82-2.07)	0.27		
**Differentiation status**		0.001		0.038
Well + Moderate	Reference	>0.99	Reference	>0.99
Poor	2.35 (1.18-4.69)	0.015	1.75 (0.84-3.64)	0.13
Undifferentiated	3.67 (1.81-7.47)	<0.001	2.62 (1.22-5.64)	0.014
**Lymph node metastasis**		<0.001		0.006
N0	Reference	>0.99	Reference	>0.99
N1	3.16 (1.35-7.44)	0.008	2.79 (1.20-6.49)	0.018
N2	3.32 (1.33-8.24)	0.01	2.92 (1.22-5.64)	0.016
N3a	6.18 (2.67-14.29)	<0.001	4.13 (1.72-9.94)	0.002
N3b	9.28 (3.94-21.87)	<0.001	5.55 (2.22-13.84)	<0.001
**Chemotherapy** (Yes vs. No)	0.75 (0.46-1.23)	0.26		

Association of all variables with peritoneal metastasis is analyzed using a two-sided Gray's test.
Abbreviations: BMI, body mass index; CA, carbohydrate antigen; CEA, carcinoembryonic antigen, CI, confidence interval; SHR, subdistribution hazard ratio.

Supplementary Table 5. C-index comparison between the two models

Model	C-index (95% CI)	*P* value
**Training Cohort**		
Nomogram	0.792 (0.784-0.798)	<0.001
Clinicopathological model	0.757 (0.748-0.765)	
**Validation Cohort**		
Nomogram	0.708 (0.692-0.726)	0.016
Clinicopathological model	0.676 (0.662-0.697)	
**Total cohort**		
Nomogram	0.779 (0.773-0.786)	<0.001
Clinicopathological model	0.736 (0.725-0.746)	

The comparisons of C-indexes between two models are performed using a two-sided Mann-Whitney *U* test.

Supplementary Table 6. AUROC comparison between the two models

Model	AUROC (95% CI)	*P* value
**Training Cohort**		
Nomogram	0.825 (0.765-0.885)	0.01
Clinicopathological model	0.787 (0.724-0.850)	
**Validation Cohort**		
Nomogram	0.776 (0.699-0.853)	0.004
Clinicopathological model	0.721 (0.635-0.807)	
**Total cohort**		
Nomogram	0.807 (0.760-0.855)	<0.001
Clinicopathological model	0.762 (0.712-0.813)	

The comparisons of AUROCs between two models are performed using a two-sided, Delong test.

Abbreviations: AUROC, area under the receiver operating characteristic curve

Supplementary Table 7. Summarization of all 146 features

No.	Feature descriptions
**Morphological features**	
1-2	Mean and variation of collagen area
3-4	Mean and variation of collagen length
5-6	Mean and variation of collagen width
7-8	Mean and variation of collagen straightness
9-10	Mean and variation of collagen cross-link density
11-12	Mean and variation of collagen orientation
**Histogram-based features**	
13-18	Mean, variance, skewness, kurtosis, energy, and entropy
**Grey -level co-occurrence matrix-based features**	
19-98	Contrast, correlation, energy and homogeneity from the grey-level co-occurrence matrix (GLCM) given five different pixel distances with four different directions
**Gabor wavelet transform features**	
99-146	Mean and variance in the convolution over the image with the Gabor filter at four scales with six orientations
