# Supplementary material for: Predicting postoperative peritoneal metastasis in gastric cancer with serosal invasion using a collagen nomogram
Source: Nat Commun. 2021 Jan 8;12:179. doi: 10.1038/s41467-020-20429-0 (PMC7794254; doi:10.1038/s41467-020-20429-0)
Supplement: Supplementary file 3 — Description of Additional Supplementary Files [file 41467_2020_20429_MOESM3_ESM.pdf]

Description of Additional Supplementary Files

File Name: Supplementary Data 1
Description: The Supplementary Data 1 indicates the source data of Table 1, the characteristics of patients in the training and validation cohorts.

File Name: Supplementary Data 2
Description: The Supplementary Data 2 indicates the source data of Table 2, the univariate and multivariate Fine-Gray regression in the training cohort.

File Name: Supplementary Data 3
Description: The Supplementary Data 3 indicates the source data of Supplementary Table 5, the C-index comparison between the two models.

File Name: Supplementary Software 1
Description: Indicates the installation guide for software and demo for data processing and analysis.
