# Supplementary material for: Predicting postoperative peritoneal metastasis in gastric cancer with serosal invasion using a collagen nomogram
Source: Nat Commun. 2021 Jan 8;12:179. doi: 10.1038/s41467-020-20429-0 (PMC7794254; doi:10.1038/s41467-020-20429-0)
Supplement: Supplementary file 7 — Supplementary Software 1 [file 41467_2020_20429_MOESM7_ESM.pdf]

Supplementary Software 1

Predicting postoperative peritoneal metastasis in gastric cancer with serosal invasion using a collagen nomogram

Authors: Dexin Chen, Zhangyuanzhu Liu, Wenju Liu, Meiting Fu, Wei Jiang, Shuoyu Xu, Guangxing Wang, Feng Chen, Jianping Lu, Hao Chen, Xiaoyu Dong, Guoxin Li, Gang Chen, Shuangmu Zhuo and Jun Yan

All associated codes used in this study are deposited in the GitHub: https://github.com/Dexin-Chen/Peritoneal_Metastasis.

1. **System requirements**

R is a **free software** environment for statistical computing and graphics. It compiles and runs on a wide variety of UNIX platforms, Windows and MacOS. The latest version of R software is available at https://www.r-project.org/.

2. **Installation guide**

The R is installed as usual based on the installation package downloaded from the https://www.r-project.org/.

Detailed procedures are provided as follows:

Step 1: Click the download R link

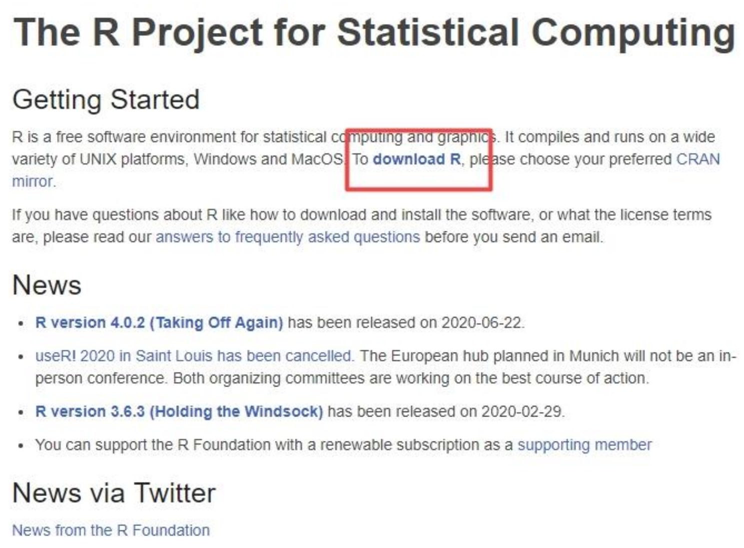

Step 2: The Comprehensive R Archive Network is available at the following URLs, please choose a location close to you.

CRAN Mirrors

The Comprehensive R Archive Network is available at the following URLs, please choose a location close to you. Some statistics on the status of the mirrors can be found here: main page, windows release, windows old release.

If you want to host a new mirror at your institution, please have a look at the CRAN Mirror HOWTO.

0-Cloud		
	https://cloud.r-project.org/	Automatic redirection to servers worldwide, currently sponsored by Rstudio
Algeria		
	https://cran.usthb.dz/	University of Science and Technology Houari Boumediene
Argentina		
	http://mirror.fcaglp.unlp.edu.ar/CRAN/	Universidad Nacional de La Plata
Australia		
	https://cran.csiro.au/	CSIRO
	https://mirror.aarnet.edu.au/pub/CRAN/	AARNET
	https://cran.ms.unimelb.edu.au/	School of Mathematics and Statistics, University of Melbourne
	https://cran.curtin.edu.au/	Curtin University
Austria		
	https://cran.wu.ac.at/	Wirtschaftsuniversität Wien
Belgium		
	https://www.freestatistics.org/cran/	Patrick Wessa
	https://lib.ugent.be/CRAN/	Ghent University Library
Brazil		
	https://nbcgib.uesc.br/mirrors/cran/	Computational Biology Center at Universidade Estadual de Santa Cruz
	https://cran-r.c3sl.ufpr.br/	Universidade Federal do Parana
	https://cran.fiocruz.br/	Oswaldo Cruz Foundation, Rio de Janeiro
	https://vps.fmvz.usp.br/CRAN/	University of Sao Paulo, Sao Paulo
	https://brieger.esalq.usp.br/CRAN/	University of Sao Paulo, Piracicaba
Bulgaria		
	https://ftp.uni-sofia.bg/CRAN/	Sofia University
Canada		
	https://mirror.rcg.sfu.ca/mirror/CRAN/	Simon Fraser University, Burnaby
	https://muug.ca/mirror/cran/	Manitoba Unix User Group
	https://mirror.its.dal.ca/cran/	Dalhousie University, Halifax
	http://cran.utstat.utoronto.ca/	University of Toronto
Chile		
	https://cran.dcc.uchile.cl/	Departamento de Ciencias de la Computación, Universidad de Chile

Step 3: Choose an operation system that is suitable for your computer.

The Comprehensive R Archive Network

Download and Install R

Precompiled binary distributions of the base system and contributed packages, **Windows and Mac** users most likely want one of these versions of R:

- Download R for Linux
- Download R for (Mac) OS X
- Download R for Windows

R is part of many Linux distributions, you should check with your Linux package management system in addition to the link above.

Source Code for all Platforms

Windows and Mac users most likely want to download the precompiled binaries listed in the upper box, not the source code. The sources have to be compiled before you can use them. If you do not know what this means, you probably do not want to do it!

- The latest release (2020-06-22, Taking Off Again) R-4.0.2.tar.gz, read what's new in the latest version.
- Sources of R alpha and beta releases (daily snapshots, created only in time periods before a planned release).
- Daily snapshots of current patched and development versions are available here. Please read about new features and bug fixes before filing corresponding feature requests or bug reports.
- Source code of older versions of R is available here.
- Contributed extension packages

Questions About R

- If you have questions about R like how to download and install the software, or what the license terms are, please read our answers to frequently asked questions before you send an email.

Step 4: Choose the 'base'.

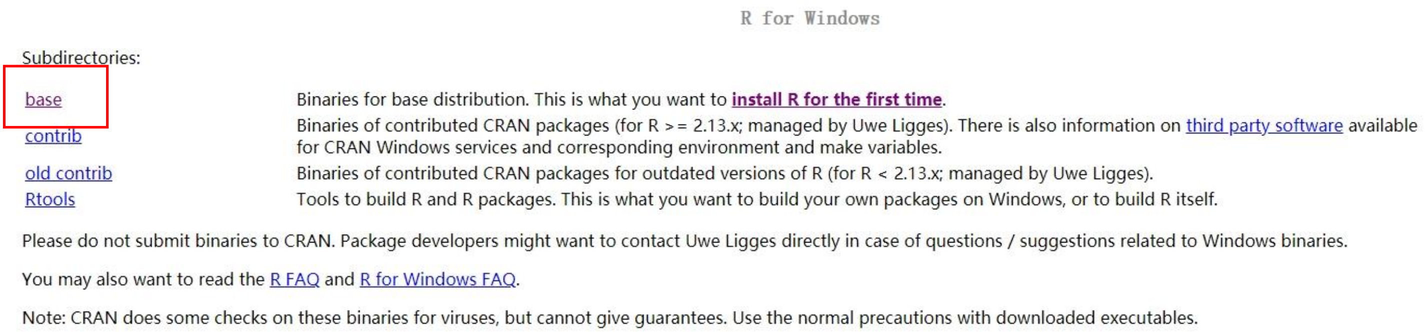
R for Windows

Subdirectories:

base	Binaries for base distribution. This is what you want to **install R for the first time**.
contrib	Binaries of contributed CRAN packages (for R >= 2.13.x; managed by Uwe Ligges). There is also information on third party software available for CRAN Windows services and corresponding environment and make variables.
old contrib	Binaries of contributed CRAN packages for outdated versions of R (for R < 2.13.x; managed by Uwe Ligges).
Rtools	Tools to build R and R packages. This is what you want to build your own packages on Windows, or to build R itself.

Please do not submit binaries to CRAN. Package developers might want to contact Uwe Ligges directly in case of questions / suggestions related to Windows binaries.

You may also want to read the R FAQ and R for Windows FAQ.

Note: CRAN does some checks on these binaries for viruses, but cannot give guarantees. Use the normal precautions with downloaded executables.

Step 5. Download the installation package.

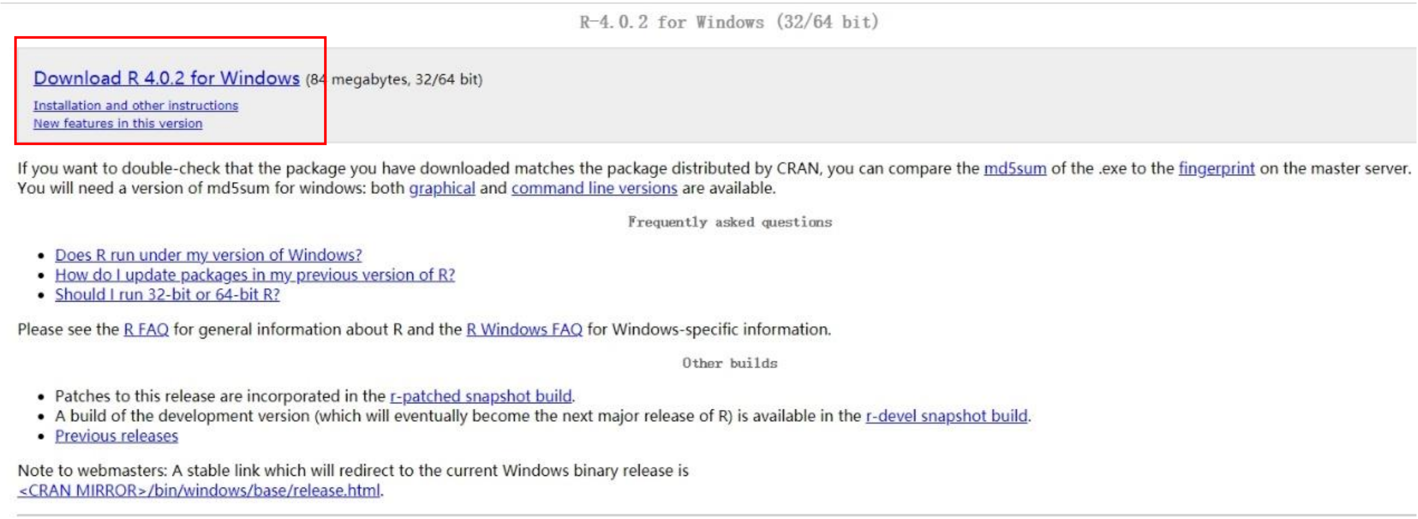
R-4.0.2 for Windows (32/64 bit)

Download R 4.0.2 for Windows (84 megabytes, 32/64 bit)

Installation and other instructions

New features in this version

If you want to double-check that the package you have downloaded matches the package distributed by CRAN, you can compare the md5sum of the .exe to the fingerprint on the master server. You will need a version of md5sum for windows: both graphical and command line versions are available.

Frequently asked questions

- Does R run under my version of Windows?
- How do I update packages in my previous version of R?
- Should I run 32-bit or 64-bit R?

Please see the R FAQ for general information about R and the R Windows FAQ for Windows-specific information.

Other builds

- Patches to this release are incorporated in the r-patched snapshot build.
- A build of the development version (which will eventually become the next major release of R) is available in the r-devel snapshot build.
- Previous releases

Note to webmasters: A stable link which will redirect to the current Windows binary release is <CRAN MIRROR>/bin/windows/base/release.html.

Meanwhile, we recommend to install Rstudio after installing R, which is an integrated development environment (IDE) for R. It includes a console, syntax-highlighting editor that supports direct code execution, as well as tools for plotting, history, debugging and workspace management, which is convenient for users to run the code.

RStudio is available in **open source** and **commercial** editions, and runs on the desktop (Windows, Mac, and Linux) or in a browser connected to RStudio Server or RStudio

Server Pro (Debian/Ubuntu, Red Hat/CentOS, and SUSE Linux). In this study, we just need the **open source** edition.

The download website is https://rstudio.com/products/rstudio/.

Step 1. When entering the website, users should drop down the page, and then select the RStudio Desktop.

Step 2. Select the open source edition, which is free for users.

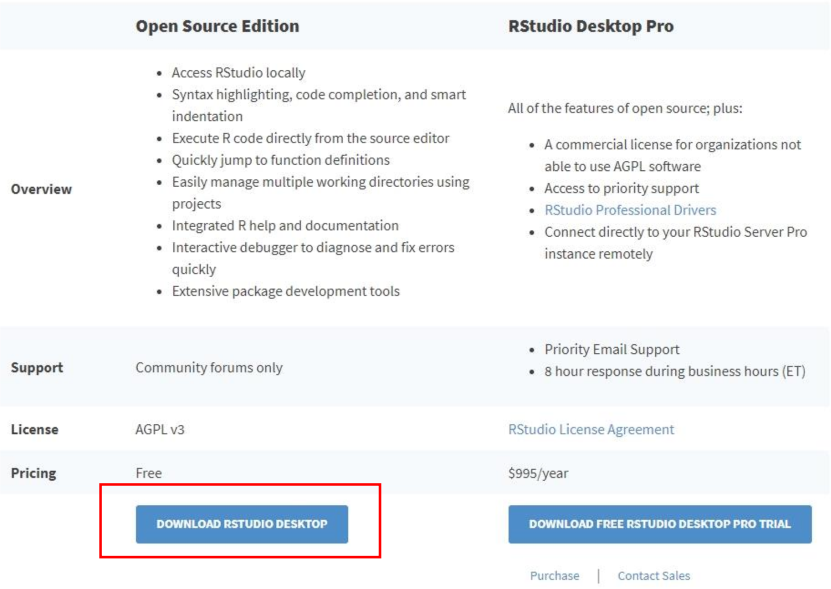

	Open Source Edition	RStudio Desktop Pro
Overview	• Access RStudio locally<br>• Syntax highlighting, code completion, and smart indentation<br>• Execute R code directly from the source editor<br>• Quickly jump to function definitions<br>• Easily manage multiple working directories using projects<br>• Integrated R help and documentation<br>• Interactive debugger to diagnose and fix errors quickly<br>• Extensive package development tools	All of the features of open source; plus:<br>• A commercial license for organizations not able to use AGPL software<br>• Access to priority support<br>• RStudio Professional Drivers<br>• Connect directly to your RStudio Server Pro instance remotely
Support	Community forums only	• Priority Email Support<br>• 8 hour response during business hours (ET)
License	AGPL v3	RStudio License Agreement
Pricing	Free	$995/year
	DOWNLOAD RSTUDIO DESKTOP	DOWNLOAD FREE RSTUDIO DESKTOP PRO TRIAL<br>Purchase | Contact Sales

Step 3: Click the DOWNLOAD.

	RStudio Desktop<br>Open Source License<br>**Free**	RStudio Desktop<br>Commercial License<br>**$995**<br>/year	RStudio Server<br>Open Source License<br>**Free**	RStudio Server Pro<br>Commercial License<br>**$4,975**<br>/year<br>(5 Named Users)
	DOWNLOAD<br>Learn more	BUY<br>Learn more	DOWNLOAD<br>Learn more	BUY<br>Evaluation | Learn more
Integrated Tools for R	□	□	□	□
Priority Support		□		□
Access via Web Browser			□	□
Enterprise Security				□
Project Sharing				□
Manage Multiple R Sessions & Versions				□
Admin Dashboard				□
Load Balancing				□
Auditing and Monitoring				□

Step 4: Select the optimal Installer. The default is recommended for Windows system.

RStudio Desktop 1.3.1073 - Release Notes

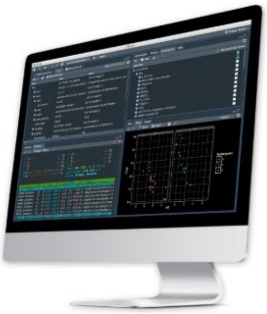

1. Install R. RStudio requires R 3.0.1+.
2. Download RStudio Desktop. Recommended for your system:

DOWNLOAD RSTUDIO FOR WINDOWS
1.3.1073 | 171.62MB

Requires Windows 10/8/7 (64-bit)

All Installers

Linux users may need to import RStudio's public code-signing key prior to installation, depending on the operating system's security policy

RStudio requires a 64-bit operating system. If you are on a 32 bit system, you can use an older version of RStudio.

OS	Download	Size	SHA-256
Windows 10/8/7	RStudio-1.3.1073.exe	171.62 MB	2fea472a
macOS 10.13+	RStudio-1.3.1073.dmg	148.66 MB	0878b305
Ubuntu 16	rstudio-1.3.1073-amd64.deb	124.07 MB	6d71c5ff
Ubuntu 18/Debian 10	rstudio-1.3.1073-amd64.deb	126.78 MB	86be9352
Fedora 19/Red Hat 7	rstudio-1.3.1073-x86_64.rpm	146.95 MB	01abb3d8

After downloading the installation package, just install it as usual.

3. Demo

We selected the Supplementary Figure 3 as an example.

Step 1: Double-click the Rstudio logo on the desktop.

Step 2: Deposit the source data and code in the same folder, and then open the R code file which you want to run.

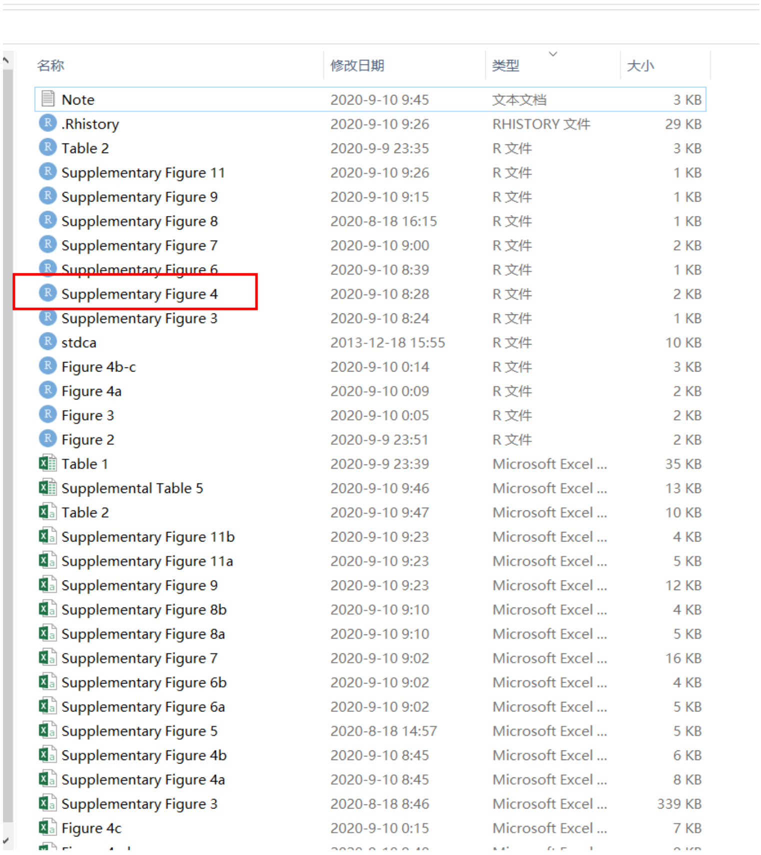

名称	修改日期	类型	大小
Note	2020-9-10 9:45	文本文档	3 KB
.Rhistory	2020-9-10 9:26	RHISTORY 文件	29 KB
Table 2	2020-9-9 23:35	R 文件	3 KB
Supplementary Figure 11	2020-9-10 9:26	R 文件	1 KB
Supplementary Figure 9	2020-9-10 9:15	R 文件	1 KB
Supplementary Figure 8	2020-8-18 16:15	R 文件	1 KB
Supplementary Figure 7	2020-9-10 9:00	R 文件	2 KB
Supplementary Figure 6	2020-9-10 8:39	R 文件	1 KB
Supplementary Figure 4	2020-9-10 8:28	R 文件	2 KB
Supplementary Figure 3	2020-9-10 8:24	R 文件	1 KB
stdca	2013-12-18 15:55	R 文件	10 KB
Figure 4b-c	2020-9-10 0:14	R 文件	3 KB
Figure 4a	2020-9-10 0:09	R 文件	2 KB
Figure 3	2020-9-10 0:05	R 文件	2 KB
Figure 2	2020-9-9 23:51	R 文件	2 KB
Table 1	2020-9-9 23:39	Microsoft Excel ...	35 KB
Supplemental Table 5	2020-9-10 9:46	Microsoft Excel ...	13 KB
Table 2	2020-9-10 9:47	Microsoft Excel ...	10 KB
Supplementary Figure 11b	2020-9-10 9:23	Microsoft Excel ...	4 KB
Supplementary Figure 11a	2020-9-10 9:23	Microsoft Excel ...	5 KB
Supplementary Figure 9	2020-9-10 9:23	Microsoft Excel ...	12 KB
Supplementary Figure 8b	2020-9-10 9:10	Microsoft Excel ...	4 KB
Supplementary Figure 8a	2020-9-10 9:10	Microsoft Excel ...	5 KB
Supplementary Figure 7	2020-9-10 9:02	Microsoft Excel ...	16 KB
Supplementary Figure 6b	2020-9-10 9:02	Microsoft Excel ...	4 KB
Supplementary Figure 6a	2020-9-10 9:02	Microsoft Excel ...	5 KB
Supplementary Figure 5	2020-8-18 14:57	Microsoft Excel ...	5 KB
Supplementary Figure 4b	2020-9-10 8:45	Microsoft Excel ...	6 KB
Supplementary Figure 4a	2020-9-10 8:45	Microsoft Excel ...	8 KB
Supplementary Figure 3	2020-8-18 8:46	Microsoft Excel ...	339 KB
Figure 4c	2020-9-10 0:15	Microsoft Excel ...	7 KB
[illegible]	[illegible]	[illegible]	[illegible]

Step 3: The functions are generally original from a specific R package. If you use a function for the first time, you should install the package first. For example, the row 1 “install.packages(“ggthemes”)”, you should put your cursor at line 1, and then click “Run” in the topright. You just need to run “library(ggthemes)” when you use the same function next time.

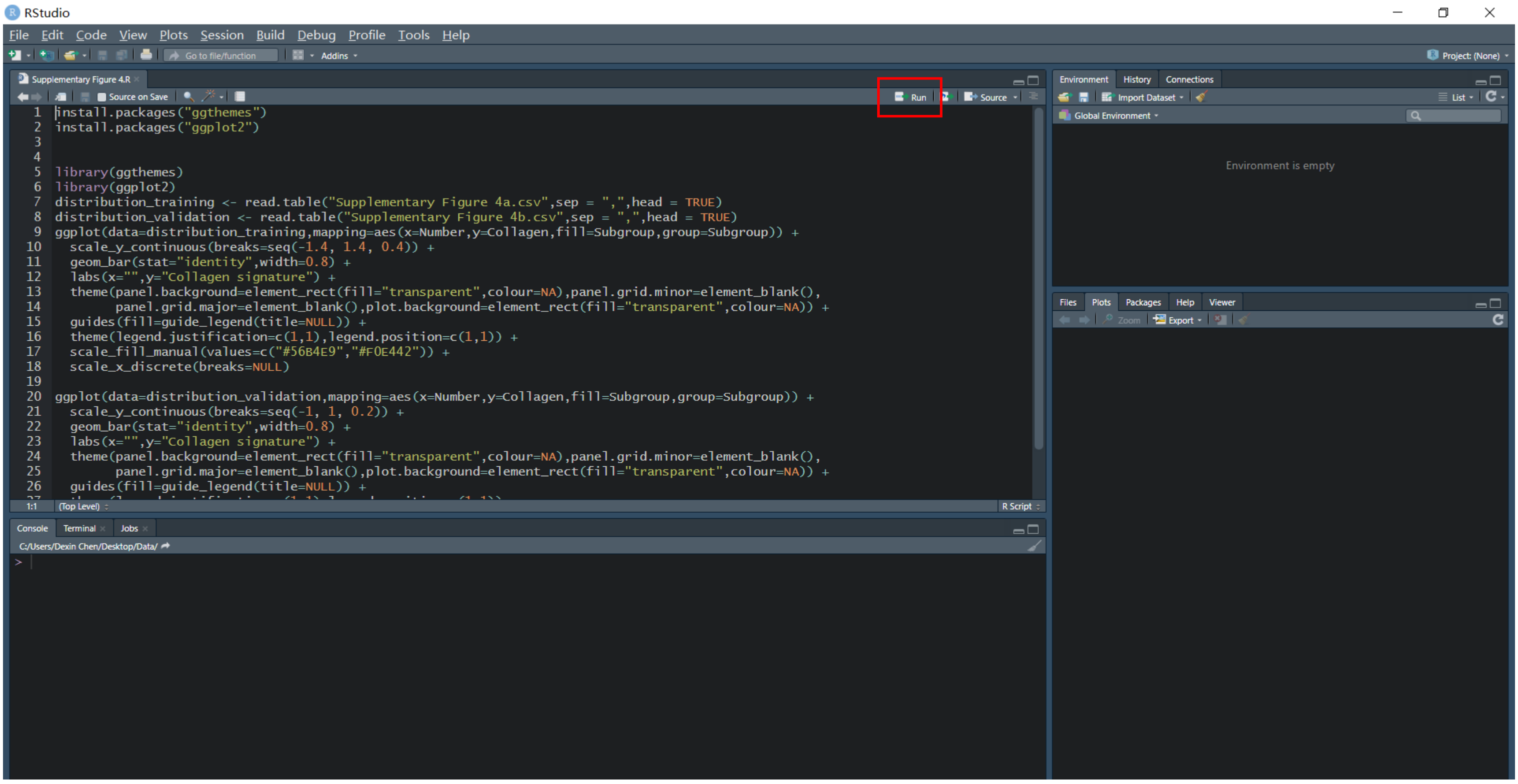

Step 4. Click the “Run” in turn, and the results including the figures and statistical analyses will be showed at the frame below.

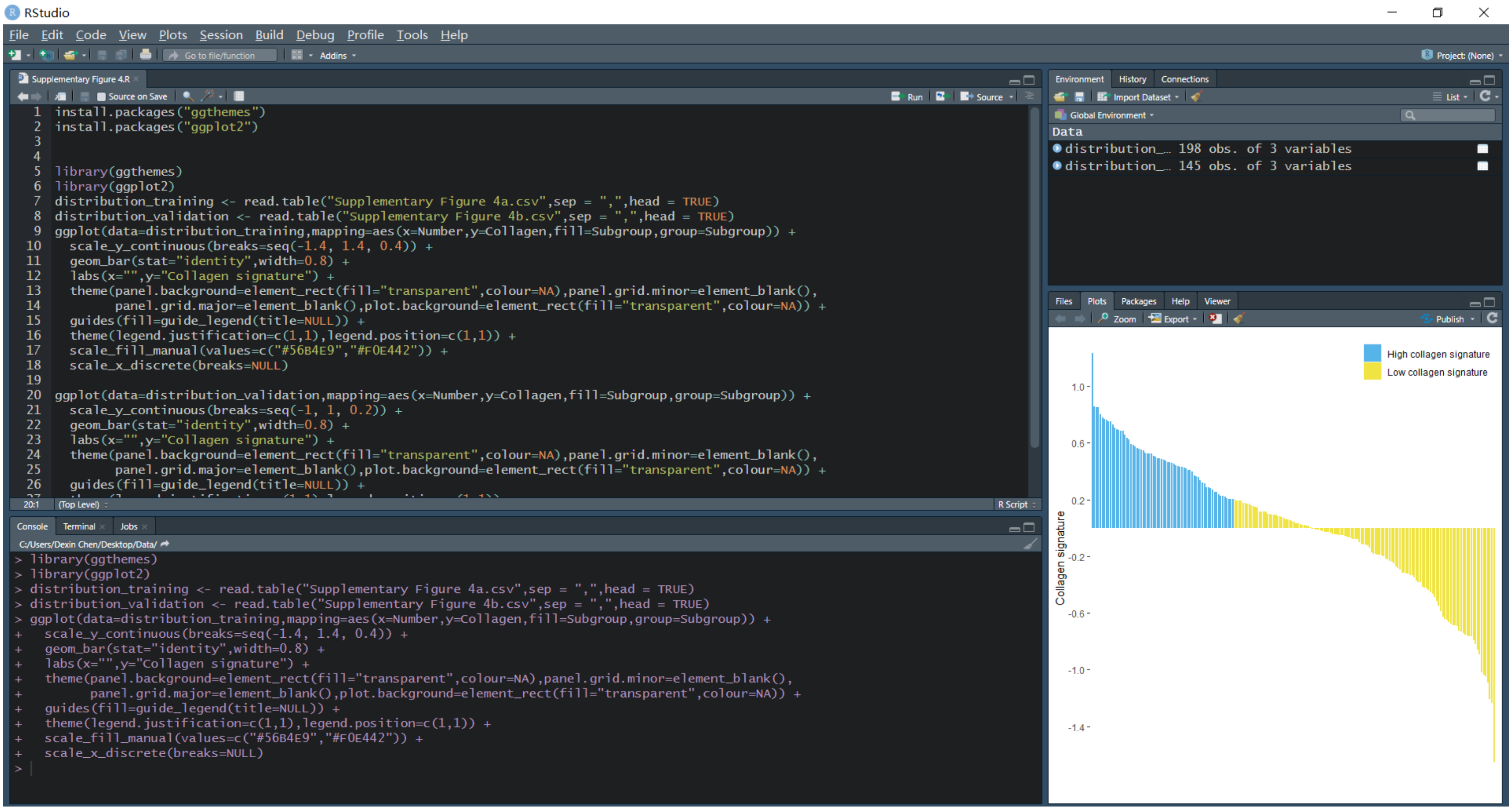

Step 5. Export the figures by click the “Export”. We recommend to export as .pdf or .eps so that we are convenient to merge or beautify the figures in Adobe Illustrator.

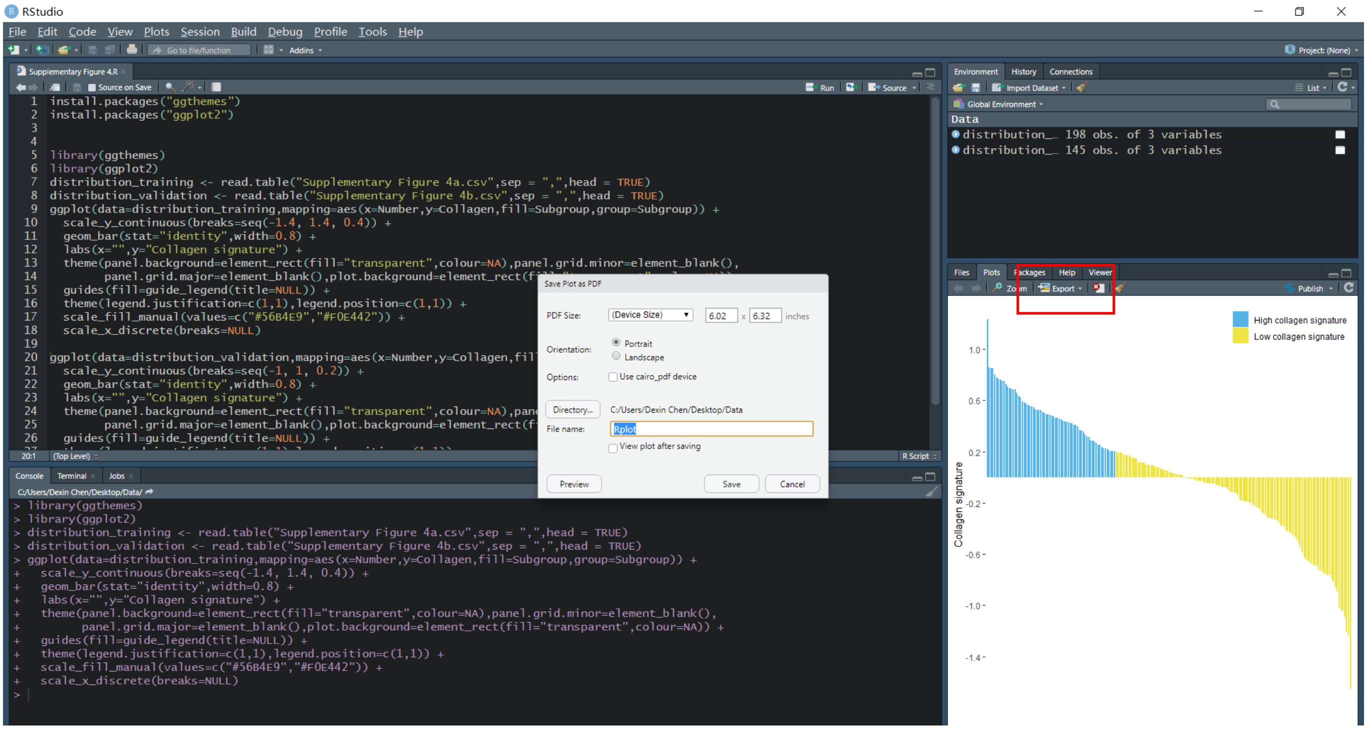
RStudio
File Edit Code View Plots Session Build Debug Profile Tools Help
Supplementary Figure 4.R
install.packages("ggthemes")
install.packages("ggplot2")
library(ggthemes)
library(ggplot2)
distribution_training <- read.table("Supplementary Figure 4a.csv",sep = ",",head = TRUE)
distribution_validation <- read.table("Supplementary Figure 4b.csv",sep = ",",head = TRUE)
ggplot(data=distribution_training,mapping=aes(x=Number,y=Collagen,fill=Subgroup,group=Subgroup)) +
scale_y_continuous(breaks=seq(-1.4, 1.4, 0.4)) +
geom_bar(stat="identity",width=0.8) +
labs(x="",y="Collagen signature") +
theme(panel.background=element_rect(fill="transparent",colour=NA),panel.grid.minor=element_blank(),
panel.grid.major=element_blank(),plot.background=element_rect(fill="transparent",colour=NA)) +
guides(fill=guide_legend(title=NULL)) +
theme(legend.justification=c(1,1),legend.position=c(1,1)) +
scale_fill_manual(values=c("#56B4E9","#F0E442")) +
scale_x_discrete(breaks=NULL)
ggplot(data=distribution_validation,mapping=aes(x=Number,y=Collagen,fil
scale_y_continuous(breaks=seq(-1, 1, 0.2)) +
geom_bar(stat="identity",width=0.8) +
labs(x="",y="Collagen signature") +
Save Plot as PDF
PDF Size: (Device Size) 6.02 x 6.32 inches
Orientation: Portrait Landscape
Options: Use cairo_pdf device
Directory... C:/Users/Dexin Chen/Desktop/Data
File name: Rplot
View plot after saving
Preview Save Cancel
Environment History Connections
Data
distribution_... 198 obs. of 3 variables
distribution_... 145 obs. of 3 variables
Files Plots Packages Help Viewer
Zoom Export Publish
High collagen signature
Low collagen signature
Collagen signature
1.0
0.6
0.2
-0.2
-0.6
-1.0
-1.4
