# Supplementary material for: Predicting postoperative peritoneal metastasis in gastric cancer with serosal invasion using a collagen nomogram
Source: Nat Commun. 2021 Jan 8;12:179. doi: 10.1038/s41467-020-20429-0 (PMC7794254; doi:10.1038/s41467-020-20429-0)
Supplement: Supplementary file 8 — Reporting Summary [file 41467_2020_20429_MOESM8_ESM.pdf]

nature research

Corresponding author(s): Jun Yan

Last updated by author(s): Nov 22, 2020

# Reporting Summary

Nature Research wishes to improve the reproducibility of the work that we publish. This form provides structure for consistency and transparency in reporting. For further information on Nature Research policies, see our Editorial Policies and the Editorial Policy Checklist.

## Statistics

For all statistical analyses, confirm that the following items are present in the figure legend, table legend, main text, or Methods section.

| n/a | Confirmed | |
|---|---|---|
| [ ] | [x] | The exact sample size (*n*) for each experimental group/condition, given as a discrete number and unit of measurement |
| [ ] | [x] | A statement on whether measurements were taken from distinct samples or whether the same sample was measured repeatedly |
| [ ] | [x] | The statistical test(s) used AND whether they are one- or two-sided<br>*Only common tests should be described solely by name; describe more complex techniques in the Methods section.* |
| [ ] | [x] | A description of all covariates tested |
| [ ] | [x] | A description of any assumptions or corrections, such as tests of normality and adjustment for multiple comparisons |
| [ ] | [x] | A full description of the statistical parameters including central tendency (e.g. means) or other basic estimates (e.g. regression coefficient) AND variation (e.g. standard deviation) or associated estimates of uncertainty (e.g. confidence intervals) |
| [ ] | [x] | For null hypothesis testing, the test statistic (e.g. *F*, *t*, *r*) with confidence intervals, effect sizes, degrees of freedom and *P* value noted<br>*Give P values as exact values whenever suitable.* |
| [x] | [ ] | For Bayesian analysis, information on the choice of priors and Markov chain Monte Carlo settings |
| [ ] | [x] | For hierarchical and complex designs, identification of the appropriate level for tests and full reporting of outcomes |
| [ ] | [x] | Estimates of effect sizes (e.g. Cohen's *d*, Pearson's *r*), indicating how they were calculated |

*Our web collection on statistics for biologists contains articles on many of the points above.*

## Software and code

Policy information about availability of computer code

| | |
|---|---|
| Data collection | The extraction of collagen features was performed automatically via MATLAB 2015b (Mathworks, Natick, MA, USA), which has previously been reported by our group (Xu S, et al., 2016, J Biophotonics). |
| Data analysis | All statistical analyses were performed using R software (version 3.4.2) and SPSS software (version 19.0). LASSO regression was performed using the "glmnet" (version 2.0-18) package. Fine-Gray competing-risk regression analysis and nomogram development were performed by the "cmprsk" (version 2.2-7.1), "rms" (version 5.1-3.1) and "mstate" (version 0.2.11) packages. Assessment of the performance and validation of the nomogram were conducted using "pec" (version 2018.07.26) and "riskRegression" (version 2019.01.29) packages. ROC curves were plotted using "pROC" (version 1.15.0) package. Decision curve analysis was performed with the function of "stdca.R". The "survminer" (version 0.4.5) package was used for computing survival analyses. Associated codes to process and analyse data are available at the GitHub: https://github.com/Dexin-Chen/Peritoneal_Metastasis. |

For manuscripts utilizing custom algorithms or software that are central to the research but not yet described in published literature, software must be made available to editors and reviewers. We strongly encourage code deposition in a community repository (e.g. GitHub). See the Nature Research guidelines for submitting code & software for further information.

## Data

Policy information about availability of data

All manuscripts must include a data availability statement. This statement should provide the following information, where applicable:

- Accession codes, unique identifiers, or web links for publicly available datasets
- A list of figures that have associated raw data
- A description of any restrictions on data availability

The source data file contains Fig. 2-4 and Supplementary Figure 3-10. The multiphoton images are not publicly available due to the laboratory regulations, but are

available from the corresponding authors upon reasonable request. The remaining data are available within the article, supplementary information or available from the corresponding authors upon request.

# Field-specific reporting

Please select the one below that is the best fit for your research. If you are not sure, read the appropriate sections before making your selection.

☒ Life sciences ☐ Behavioural & social sciences ☐ Ecological, evolutionary & environmental sciences

For a reference copy of the document with all sections, see nature.com/documents/nr-reporting-summary-flat.pdf

# Life sciences study design

All studies must disclose on these points even when the disclosure is negative.

| | |
|---|---|
| Sample size | No sample-size calculation was performed. For developing a competing-risk prediction model, consensus of sample-size calculation has not yet been reached. According to the TRIPOD Statement (Moons et al., 2015, Ann Intern Med), at least 10 outcome events per variable (EPV) was needed. However, as the Statement noted, the 10 EPV was based on two empirical investigations (Peduzzi et al., 1995, J Clin Epidemiol; Peduzzi et al., 1996, J Clin Epidemiol). Some researches suspected that the 10 EPV is too lenient (Wynants et al., 2015, J Clin Epiermiol), or too strict (Vittinghoff et al., 2007, Am J Epidemiol). Only for a planned prospective prediction model development study will the sample size be predetermined on statistical grounds (Karel et al., 2015, Ann Intern Med). In this study, a total of 4 variables were included to develop the nomogram. There were 79 patients suffered from peritoneal metastasis after radical surgery (more than 40), which was enough. For sample size calculation in the validation cohort, a previous study, Lei et al reported that the ratio between training and validation was 7:3 (Lei et al., 2015, JAMA Surgery). In our study, the validation cohort contained 145 patients, which was also adequate. |
| Data exclusions | We excluded patients with neoadjuvant radiotherapy, neoadjuvant chemotherapy or neoadjuvant chemoradiotherapy. Because anticancer therapy before radical surgery could affect the oncological outcomes. The exclusion criteria were pre-established before conducting data collection and analysis. |
| Replication | After the development of the competing-risk nomogram, the validation cohort was used to verify the reproducibility of the predictive performance of the nomogram. All attempts at replication were successful. |
| Randomization | The purpose of this study was to develop and validate a competing-risk nomogram to predict the risk of peritoneal metastasis. Both the training and validation cohorts were consecutive patients, therefore, randomization was not needed. |
| Blinding | Two independent pathologists were blinded to clinical characteristics and prognosis when reassessing the invasive region of the gastric serosa. |

# Reporting for specific materials, systems and methods

We require information from authors about some types of materials, experimental systems and methods used in many studies. Here, indicate whether each material, system or method listed is relevant to your study. If you are not sure if a list item applies to your research, read the appropriate section before selecting a response.

## Materials & experimental systems

| n/a | Involved in the study | |
|---|---|---|
| ☒ | ☐ | Antibodies |
| ☒ | ☐ | Eukaryotic cell lines |
| ☒ | ☐ | Palaeontology and archaeology |
| ☒ | ☐ | Animals and other organisms |
| ☐ | ☒ | Human research participants |
| ☒ | ☐ | Clinical data |
| ☒ | ☐ | Dual use research of concern |

## Methods

| n/a | Involved in the study | |
|---|---|---|
| ☒ | ☐ | ChIP-seq |
| ☒ | ☐ | Flow cytometry |
| ☒ | ☐ | MRI-based neuroimaging |

## Human research participants

Policy information about studies involving human research participants

| | |
|---|---|
| Population characteristics | Of the 198 patients in the training cohort, the median age [interquartile range (IQR)] was 57 (47.75-63.25) years, with 137 (69.2%) men. Among the 145 patients in the validation cohort, the median age (IQR) was 57 (52-64) years, with 98 (67.6%) men. There was no significant difference between the training and validation cohorts. The detailed characteristics of population is listed in Table 1. |
| Recruitment | The training cohort, including 198 consecutive patients and was obtained from the Nanfang Hospital of Southern Medical University between July 1, 2011, and July 31, 2014. The validation cohort comprising 145 consecutive patients was obtained from the Fujian Provincial Cancer Hospital of Fujian Medical University between July 1, 2008, and March 31, 2011. Inclusion criteria were patients who underwent radical gastrectomy with negative peritoneal lavage cytology and with histologically diagnosed GC with serosal invasion, the availability of clinicopathological data and a complete 3-year postoperative follow-up. We excluded patients with neoadjuvant radiotherapy, neoadjuvant chemotherapy or neoadjuvant chemoradiotherapy. In this retrospective study, all consecutive patients were enrolled strictly according to the inclusion and exclusion criteria, thus potential self selection bias or other biases were reduced markedly, which were unlikely to impact the results. |
| Ethics oversight | Institutional Review Board at Nanfang Hospital of Southern Medical University and Fujian Provincial Cancer Hospital of Fujian Medical University |

Note that full information on the approval of the study protocol must also be provided in the manuscript.
